# Incorporating Epidemiological Data into the Genomic Analysis of Partially Sampled Infectious Disease Outbreaks

**DOI:** 10.1093/molbev/msaf083

**Published:** 2025-04-21

**Authors:** Jake Carson, Matt Keeling, Paolo Ribeca, Xavier Didelot

**Affiliations:** Mathematics Institute, University of Warwick, Coventry CV4 7AL, UK; School of Life Sciences, University of Warwick, Coventry CV4 7AL, UK; Mathematics Institute, University of Warwick, Coventry CV4 7AL, UK; School of Life Sciences, University of Warwick, Coventry CV4 7AL, UK; Clinical and Emerging Infection, UK Health Security Agency, London NW9 5EQ, UK; School of Life Sciences, University of Warwick, Coventry CV4 7AL, UK; Department of Statistics, University of Warwick, Coventry CV4 7AL, UK

**Keywords:** genomic epidemiology, transmission analysis, infectious disease outbreak, epidemiological data

## Abstract

Pathogen genomic data are increasingly being used to investigate transmission dynamics in infectious disease outbreaks. Combining genomic data with epidemiological data should substantially increase our understanding of outbreaks, but this is highly challenging when the outbreak under study is only partially sampled, so that both genomic and epidemiological data are missing for intermediate links in the transmission chains. Here, we present a new dynamic programming algorithm to perform this task efficiently. We implement this methodology into the well-established TransPhylo framework to reconstruct partially sampled outbreaks using a combination of genomic and epidemiological data. We use simulated datasets to show that including epidemiological data can improve the accuracy of the inferred transmission links compared with inference based on genomic data only. This also allows us to estimate parameters specific to the epidemiological data (such as transmission rates between particular groups), which would otherwise not be possible. We then apply these methods to two real-world examples. First, we use genomic data from an outbreak of tuberculosis in Argentina, for which data was also available on the HIV status of sampled individuals, in order to investigate the role of HIV coinfection in the spread of this tuberculosis outbreak. Second, we use genomic and geographical data from the 2003 epidemic of avian influenza H7N7 in the Netherlands to reconstruct its spatial epidemiology. In both cases, we show that incorporating epidemiological data into the genomic analysis allows us to investigate the role of epidemiological properties in the spread of infectious diseases.

## Introduction

Over the past decade, there has been considerable research interest and methodological development in the analysis of pathogen genomic sequences to reconstruct the transmission events that occurred during an infectious disease outbreak (Jombart et al. 2014; Croucher and Didelot 2015; Campbell et al. 2018; Duault et al. 2022). Additional epidemiological data about the infected hosts are often available, and it can be useful to integrate such data into the genomic analysis for two complementary reasons. First, it should allow the transmission trees to be reconstructed more precisely when using genomic and epidemiological data compared with using genomic data alone. An example of this was provided by the reconstruction of a tuberculosis outbreak in British Columbia, in which the matrix of who-infected-whom probabilities contained less uncertainty when geographical data and measures of infectiousness (provided by smear and skin tests) were used as additional input (Didelot et al. 2014; Biek et al. 2015; Hatherell et al. 2016). Second, using data on epidemiological properties can enable inference on the correlation between transmission and these epidemiological properties. For example, individuals with high-risk behaviors contribute disproportionately to the spread of sexual diseases such as gonorrhoea (Chan et al. 2012; Fingerhuth et al. 2016; Whittles et al. 2019). Integrating behavioral data into a transmission analysis could help quantify this effect, which could be used in predictive models for example to inform the design of control measures such as targeted vaccination (Craig et al. 2015; Whittles et al. 2020, 2022).

Previous attempts have been made to integrate epidemiological and genomic data into outbreak reconstructions. The simplest case occurs if we assume that all cases of the outbreak have been sampled and are therefore present in the transmission tree. In this case, the likelihood of the genomic data can simply be multiplied by the likelihood of the epidemiological data (Morelli et al. 2012; Ypma et al. 2012; Didelot et al. 2014; Hall et al. 2015). The epidemiological component of the likelihood is easy to compute as a product over all links in the transmission tree (Ypma et al. 2012). Each link represents an infection from a sampled infector to a sampled infected, and as epidemiological data are available for both hosts, the contribution to the likelihood is analytically tractable. However, the vast majority of infectious disease outbreaks are only partially observed, with the proportion of missing cases being typically unknown as well (O’Neill and Roberts 1999; Chis Ster et al. 2009; Jewell et al. 2009). These missing intermediates in the transmission trees represent a challenge for the integration of epidemiological data, since by definition there is no epidemiological data available on unknown putative hosts. This difficulty was noted, for example, when a spatial-genetic framework assuming complete sampling (Morelli et al. 2012) was extended to handle incomplete sampling, with two extreme scenarios proposed as bounds on the probability of spatial dispersion for unknown cases (Mollentze et al. 2014).

A naive approach to incorporate epidemiological data into the transmission analysis of a partially sampled outbreak is to consider all possible combinations for the epidemiological data of the unsampled cases along with their associated probabilities. For each combination, the epidemiological component of the likelihood can be calculated as previously described in the case of a fully sampled outbreak (Morelli et al. 2012; Ypma et al. 2012; Didelot et al. 2014; Hall et al. 2015). The unconditioned likelihood is then be obtained as the average of these conditioned likelihoods, weighted according to their probabilities, using the law of total probability. However, the number of combinations scales exponentially with the number of unsampled cases in the transmission tree, and is only be computationally feasible for very small outbreaks. Another approach is to rely on data augmentation techniques within a Markov chain Monte Carlo (MCMC) framework, in order to treat the epidemiological data of unsampled cases as additional parameters (van Dyk and Meng 2001; O’Neill 2002). Again, this may not scale well to larger outbreaks, especially since the number of unsampled cases is unknown, so that efficient reversible jump proposals are required to deal with the transdimensional parameter space (Green 1995; Sisson 2005). Instead, we present a computationally efficient approach to calculate the epidemiological component of the likelihood. We show that this computation can be used to incorporate epidemiological data into the transmission analysis, boosting the accuracy of the analysis and generating the type of who-acquires-infection-from-whom matrices that are the cornerstone of predictive modeling. We illustrate our method on simulated datasets, before considering real-world examples of tuberculosis and H7N7 outbreaks. By incorporating epidemiological data into the genomic analysis of outbreaks, we can improve the reconstruction of transmission links and quantify the role of epidemiological properties in the spread of infectious diseases.

## New Approaches

We take as our starting point the TransPhylo methodology (Didelot et al. 2014), which represents the transmission tree by coloring the branches of an input dated phylogeny (Rieux and Balloux 2016). This dated phylogeny is assumed known, and remains fixed throughout the analysis. The first version of TransPhylo considered only fully sampled outbreaks, so that it was possible to incorporate epidemiological data (Didelot et al. 2014). With the extension of TransPhylo to the more generally useful situation of a partially sampled outbreak, this possibility to integrate epidemiological data was lost (Didelot et al. 2017, 2021). More recently, TransPhylo was further extended to allow some hosts to be sampled more than once and to remove the assumption of complete transmission bottleneck (Carson et al. 2024), and this is the version that we use as our starting point for the incorporation of epidemiological data.

We extend the TransPhylo framework to incorporate known discrete epidemiological data on the sampled hosts, or a subset of them. Note that we use the term “deme” to represent data that could be any discrete property of the hosts, for example, geographical location in different towns or hospital wards, age, or gender categories, classification based on behavioral data, infectious status from other infectious diseases, etc. We let *S* denote the number of demes (number of discrete epidemiological states). The transmission model within TransPhylo is a continuous time branching process (Farrington et al. 2003), in which each infected host generates a number of offspring *k* from an offspring distribution function α(k), and their infection times *τ* relative to the infection time of the infector are sampled from a generation time distribution γ(τ). The mean of the offspring distribution is the basic reproduction number *R*. We extend this branching process so that a deme is sampled for each offspring conditional on the deme of the infector. Specifically, the probability that a newly infected host belongs to deme *j* given that their infector belongs to deme *i* is denoted $P_{ij}$. This matrix may take any form, as long as each of the rows sums up to one, and may include some parameters that we wish to infer jointly with the transmission tree. In our analyses, we make the simplifying assumption when transmissions exit a deme, their destinations are uniformly distributed across the other demes. This choice means that we only need to estimate the diagonal elements of *P*, and so the number of parameters grows linearly in the number of demes, rather than quadratically.

We consider two complementary cases. In the first case, hosts in every deme have the same offspring distribution function and probability of being sampled. Since the transmission model is independent of the deme of each host, the likelihood can be decomposed as the product of the transmission tree likelihood and the likelihood of the epidemiological data. The former can be computed as in previous versions of TransPhylo (Didelot et al. 2017; Carson et al. 2024), and we show how the latter can be calculated efficiently using a dynamic programming algorithm similar to the Felsenstein pruning algorithm (Felsenstein 1973, 1981). That is, we work backward from the tips of the phylogenetic tree, in order to efficiently integrate over all possible demes for unsampled hosts. In the second case, the offspring distribution function and probability of being sampled depend on the deme. This dependency may involve some parameters that we wish to estimate, for example different values $R_{1},\ldots ,R_{S}$ for the basic reproduction number within each of the demes. Since the transmission model now depends on the deme of each host, the likelihood can no longer be decomposed as previously. Instead, we must consider the transmission tree and epidemiological data jointly. We show that the likelihood can still be calculated analytically using a more complicated dynamic programming algorithm. This algorithm remains similar to the Felsenstein pruning algorithm, but now calculates a single likelihood for the combined transmission tree and epidemiological data.

## Results

### Exemplary Analysis of a Simulated Dataset Where All Demes Have the Same Offspring Distribution and Sampling Probabilities

**Fig. 1.**
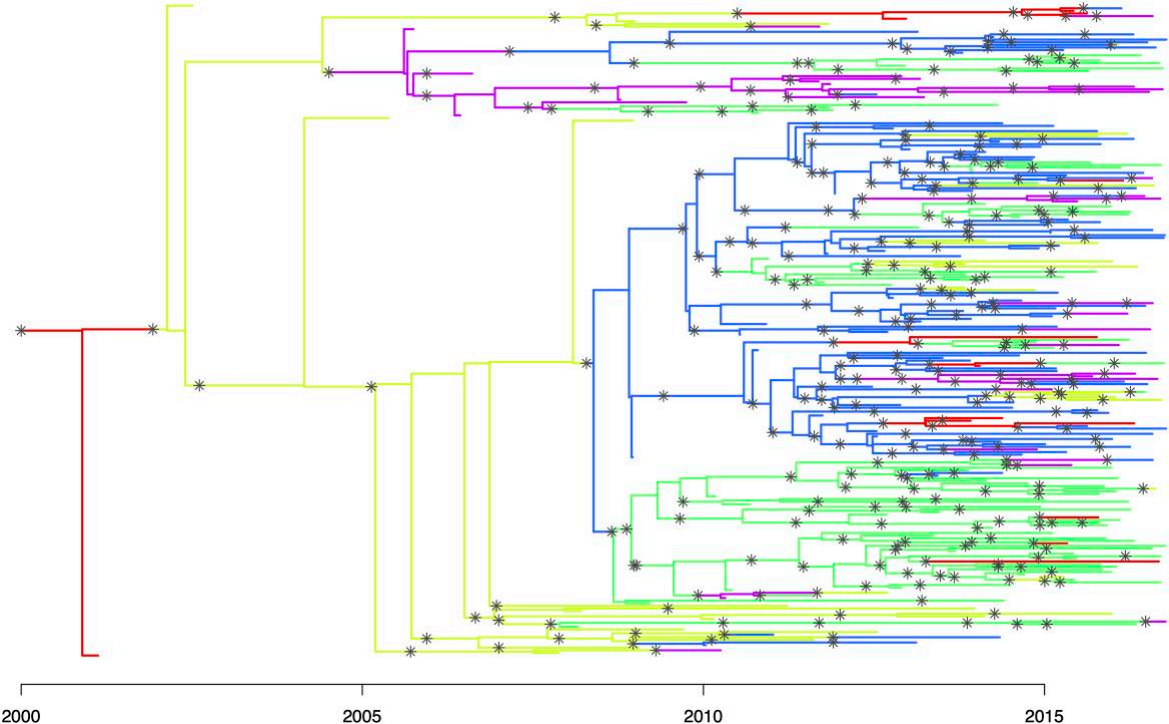
Combined transmission and phylogenetic tree for the first simulation study. The tips of the tree are samples taken from infected hosts. The tree topology provides the dated phylogenetic tree, including the date of the most recent common ancestor for any set of samples. The transmission tree is added to the phylogenetic tree through coloring. Asterisks show transmission events, separating different hosts. With a relaxed bottleneck, multiple synchronous asterisks may represent a single transmission event involving multiple lineages. Hosts are colored according to their deme. Some hosts are unsampled, contributing no tips to the phylogenetic tree. The tree contains 302 infected hosts, of which 250 are sampled.

We simulate an outbreak with 250 observed infected hosts across five demes, with each observed host being sampled once. The observation cutoff time *T* is determined by the simulation in order to return the correct number of observed infected hosts. The generation time and primary observation time are both Gamma-distributed with shape and scale parameters equal to 2 and 1, respectively. For the transmission model, the offspring distribution follows a negative binomial distribution with r=2 and p=0.5, so the basic reproduction number R=r=2. The sampling proportion is π=0.8. The within-host pathogen population size is κ+λτ at time *τ* after infection, with κ=0.1 and λ=0.2. The probability of an offspring having the same deme as their infector is ρ=0.8, otherwise one of the other four demes is sampled uniformly. The resulting simulation contains 302 infected hosts (of which 250 are sampled). The transmission and phylogenetic trees are shown in Fig. 1, which is colored according to the demes of the hosts. Note that only the deme data for the observed hosts are used in the analysis.

Our goals are to estimate the five parameters *R*, *π*, *ρ*, κ, and *λ*, and to correctly identify transmission links between sampled hosts. We generated four separate MCMC chains of 100,000 iterations, each of which took approximately 36 h on a 3 GHz processor core. The effective sample size was ¿300 for all parameters in each chain, and the multivariate Gelman–Rubin statistic comparing chains was 1.01 (Brooks and Gelman 1998). The inferred means and 95% credible intervals for each parameter are shown in the “Original” column of Table 1. This shows that we are able to recover the simulated parameter values effectively, since the posterior means are close to the correct values and the credible intervals cover the correct values.

**Table 1:** Estimated parameter values for the first simulation study

Parameter	Original	Extra obs	Noisy 5	Noisy 10	Noisy 20	No demes
R(2.0)	1.94	1.92	1.94	1.94	1.96	1.94
	(1.68,2.22)	(1.65,2.20)	(1.67,2.21)	(1.68,2.22)	(1.70,2.24)	(1.67,2.22)
π(0.8)	0.78	0.86	0.77	0.79	0.75	0.77
	(0.63,0.93)	(0.74,0.96)	(0.63,0.93)	(0.64,94)	(0.61,0.90)	(0.62,0.92)
ρ(0.8)	0.78	0.78	0.74	0.69	0.60	–
	(0.73,0.83)	(0.72,0.83)	(0.68,0.79)	(0.63,0.75)	(0.53,0.67)	–
κ(0.1)	0.09	0.09	0.11	0.11	0.10	0.12
	(0.00,0.21)	(0.06,0.13)	(0.01,0.23)	(0.02,0.25)	(0.01,0.24)	(0.01,0.25)
λ(0.2)	0.20	0.22	0.17	0.18	0.17	0.15
	(0.02,0.41)	(0.19,0.26)	(0.01,0.40)	(0.02,0.41)	(0.01,0.40)	(0.01,0.37)

The leftmost column indicates the parameter values used in the simulation. Other columns provide the posterior means and 95% credible intervals. We show results for the original simulated dataset (Original), using five samples per host (Extra obs), mislabeling 5%, 10%, and 20% of host demes (Noisy 5, Noisy 10, Noisy 20), and discarding deme information completely (No demes).

To evaluate our ability to reconstruct transmission links, we focus on transmissions between observed hosts. Out of the 250 observed hosts, 184 are infected by another observed host. If we define 0.5 as the posterior probability threshold for a transmission event being identified, we correctly identify 71 transmission links including the direction of transmission, giving a directional sensitivity of 39%. With only one observation per host it is common to identify a transmission link between two hosts, but be unsure of the direction of transmission (Didelot et al. 2014, 2017; Carson et al. 2024). If we ignore the direction of transmission we identify 104 transmission links, giving a nondirectional sensitivity of 57%. We incorrectly establish 30 directional transmission links, and 37 nondirectional transmission links. However, as there are 62,250 possible host combinations, specificity is high (>99.9%) in both cases. The resulting precision is 70% when including the direction of transmission, and 74% when ignoring the direction of transmission. This information is summarized in the “Original” column of Table 2.

**Table 2:** Performance measures for the first simulation study

Performance measure	Original	Extra obs	Noisy 5	Noisy 10	Noisy 20	No demes
Sensitivity (dir.)	39%	53%	36%	35%	34%	33%
Sensitivity (non-dir.)	57%	68%	54%	55%	52%	47%
Specificity (dir.)	>99.9%	>99.9%	>99.9%	>99.9%	>99.9%	>99.9%
Specificity (non-dir.)	>99.9%	>99.9%	>99.9%	>99.9%	>99.9%	>99.9%
Precision (dir.)	70%	71%	66%	63%	69%	71%
Precision (non-dir.)	74%	74%	71%	71%	75%	73%

We evaluate the sensitivity, specificity, and precision for both directional transmission links (dir.) and nondirectional transmission links (non-dir.). We show results for the original simulated dataset (Original), using five samples per host (Extra obs), mislabeling 5%, 10%, and 20% of host demes (Noisy 5, Noisy 10, Noisy 20), and discarding deme information completely (No demes).

If inference is undertaken without the deme data we obtain similar parameter estimates, as shown in the “No demes” column of Table 1. Note that we no longer obtain an estimate for *ρ*, as with no deme data, we default to the assumption that all hosts belong to a single deme, i.e. all hosts are treated homogeneously. The performance measures are summarized in Table 2. Compared with the analysis using the deme data, we obtain a lower sensitivity for both directional and nondirectional transmission links, but the precision is similar.

**Fig. 2.**
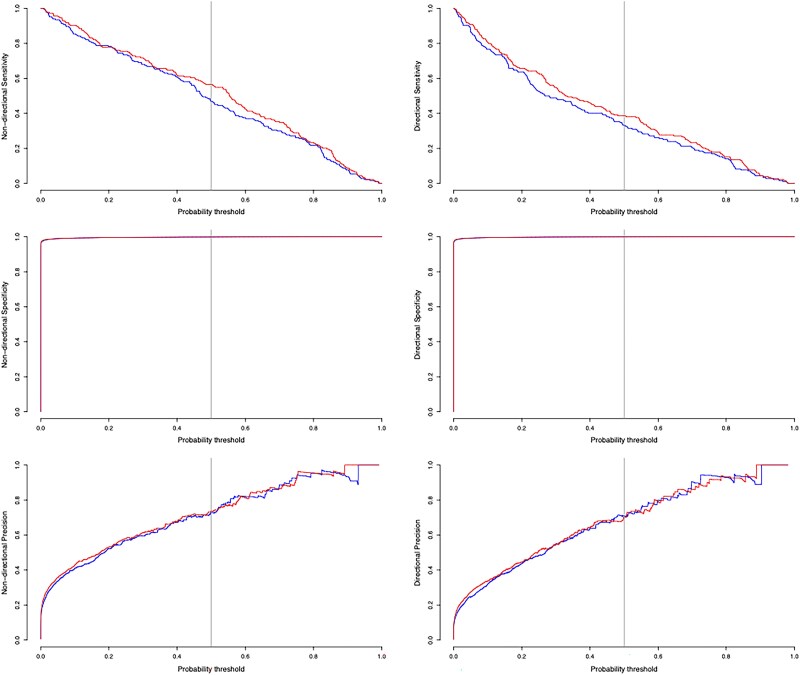
Performance measures for the first simulation study vs the probability threshold used to identify transmission links. Top: Sensitivity, middle: specificity, bottom: precision. On the left, we show nondirectional transmission links, and on the right, we show directional transmission links. The red curves indicate the performance when using deme data, and the blue curves indicate performance when ignoring deme data. The vertical line at 0.5 shows the probability threshold used in our analysis.

Figure 2 compares the performance measures as we alter the posterior probability threshold for identifying transmission links. Generally, as we increase the threshold, we obtain a lower sensitivity, but a greater precision. Using the deme information increases the sensitivity across a wide range of threshold values, but does not significantly alter the precision.

In real-world situations, it may be the case that some hosts are recorded as belonging to an incorrect deme. To test the robustness of our approach to such errors, we repeat the analysis after assigning 5%, 10%, and 20% of the observed hosts to a random incorrect deme. We compare parameter estimates in Table 1 and performance measures in Table 2, under the “Noisy 5,” “Noisy 10,” and “Noisy 20” columns, respectively. The estimates of *R*, *π*, *κ*, and *λ* remain consistent, as the deme data are not informative for these parameters. The estimate of *ρ* decreases as more errors are added, likely as more transmissions across demes are inferred from the corrupted data. The sensitivity seems to decrease as errors are added, but the precision seems to drop with 5% and 10% errors, before rebounding with 20% errors. It may be the case that with 5% and 10% errors, the estimate of *ρ* is sufficiently high that the deme information strongly influences transmission links. With corrupted deme data, we are more likely to identify incorrect transmission links. With 20% errors, the estimate of *ρ* is approaching 0.5 (completely random mixing between demes), and so the deme data are essentially ignored when establishing transmission links. In the Discussion, we consider how the algorithm could be modified to account for imperfect deme data.

Finally, to highlight the benefits of additional samples from infected hosts, we repeat the analysis with five samples per host, with additional samples being separated by fixed time intervals. We compare parameter estimations in Table 1 and performance measures in Table 2 under the “Extra obs” column. Most notably we obtain more constrained estimates for *κ* and *λ*, as well as better sensitivity for both directional and nondirectional transmission links. We also obtain a higher estimate of *π*, but the true value remains within the 95% credible interval.

### Exemplary Analysis of a Simulated Dataset Where Demes Have Different Offspring Distributions and Sampling Probabilities

**Fig. 3.**
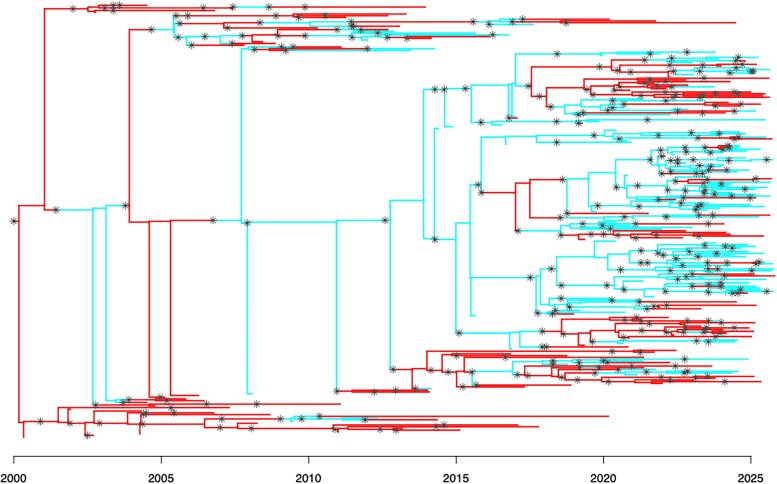
Combined transmission and phylogenetic tree for the second simulation study. The tips of the tree are samples taken from infected hosts. The tree topology provides the dated phylogenetic tree, i.e. providing the date of the most recent common ancestor for any set of samples. The transmission tree is added to the phylogenetic tree through coloring. Asterisks show transmission events, separating different hosts. With a relaxed bottleneck, multiple synchronous asterisks may represent a single transmission event involving multiple lineages. Hosts are colored according to their deme. Some hosts are unsampled, contributing no tips to the phylogenetic tree. The tree contains 325 infected hosts, of which 250 are sampled.

We simulate a second outbreak with 250 observed hosts. The hosts belong to two demes, with each deme having its own *R*, *π*, and *ρ* parameters. Specifically, we set $R_{1}=1.2$, $R_{2}=2.2$, $\pi_{1}=0.4$, $\pi_{2}=0.9$, $\rho_{1}=0.9$, $\rho_{2}=0.7$, while maintaining κ=0.1 and λ=0.2. Consequently, deme 1 has a low transmission rate and is poorly surveyed, whilst deme 2 has a high transmission rate and is well surveyed. With this choice of *ρ* values, offspring are more likely than not to be in the same deme as the infecting host, but transmissions from deme 2 to deme 1 are more likely than transmissions from deme 1 to deme 2. Specifically, we expect 1 in 10 transmissions originating from a host in deme 1 to infect a host in deme 2, whereas we expect 3 in 10 transmissions originating from a host in deme 2 to infect a host in deme 1. The resulting simulation contains 325 hosts, and the transmission and phylogenetic trees are shown in Fig. 3, which is colored according to the demes.

We generate four separate MCMC chains of 100,000 iterations, each of which took approximately 46 h on a 3 GHz processor core. The effective sample size was >300 for all parameters in each chain, and the multivariate Gelman–Rubin statistic comparing chains was 1.01 (Brooks and Gelman 1998). The inferred means and 95% credible intervals for each parameter are shown in the “Original” column of Table 3. Once again this shows that we are able to recover the simulated parameter values effectively since the inferred values are close to the correct values used in the simulation. Furthermore, since the credible intervals for $R_{1}$ and $R_{2}$, and for $\pi_{1}$ and $\pi_{2}$ do not overlap, we can deduce that we have correctly inferred that the reproduction number and sampling probability are both higher in the second deme than in the first deme.

**Table 3:** Estimated parameter values for the second simulation study

Parameter	Original	Extra obs	Noisy 5	Noisy 10	Noisy 20	No demes
$R_{1}\;(1.2)$	1.15	1.19	1.11	1.18	1.21	1.56
	(0.92,1.39)	(0.98,1.42)	(0.88,1.36)	(0.92,1.44)	(0.91,1.51)	(1.35,1.79)
$R_{2}\;(2.2)$	2.21	2.11	2.28	2.19	2.04	–
	(1.78,2.68)	(1.72,2.55)	(1.84,2.79)	(1.73,2.71)	(1.60,2.56)	–
$\pi_{1}\;(0.4)$	0.50	0.48	0.49	0.54	0.57	0.73
	(0.35,0.68)	(0.35,0.61)	(0.34,0.68)	(0.37,0.75)	(0.38,86)	(0.58,0.9)
$\pi_{2}\;(0.9)$	0.88	0.86	0.87	0.86	0.85	–
	(0.68,0.99)	(0.71,0.98)	(0.67,0.99)	(0.64,0.99)	(0.60,0.99)	–
$\rho_{1}\;(0.9)$	0.93	0.92	0.90	0.84	0.74	–
	(0.87,0.96)	(0.87,0.96)	(0.83,0.95)	(0.75,0.91)	(0.57,0.85)	–
$\rho_{2}\;(0.7)$	0.72	0.73	0.66	0.62	0.57	–
	(0.62,0.81)	(0.63,0.81)	(0.55,0.76)	(0.50,0.73)	(0.43,0.70)	–
κ(0.1)	0.10	0.08	0.09	0.12	0.10	0.09
	(0.00,0.28)	(0.04,0.12)	(0.00,0.26)	(0.01,0.29)	(0.00,0.29)	(0.00,0.25)
λ(0.2)	0.23	0.21	0.25	0.22	0.24	0.28
	(0.03,0.48)	(0.18,0.24)	(0.04,0.49)	(0.02,0.52)	(0.03,0.51)	(0.04,0.58)

The leftmost column indicates the parameter values used in the simulation. Other columns provide the posterior means and 95% credible intervals. We show results for the original simulated dataset (Original), using five samples per host (Extra obs), mislabeling 5%, 10%, and 20% of host demes (Noisy 5, Noisy 10, Noisy 20), and discarding deme information completely (No demes).

Looking at the inferred transmission links, out of the 250 observed hosts 155 are infected by another observed host. Using a posterior probability threshold of 0.5 as in the first simulation study, we correctly identify 65 transmission links including the direction of transmission, giving a directional sensitivity of 42%. If we ignore the direction of transmission then we identify 89 transmission links, giving a nondirectional sensitivity of 57%. We incorrectly establish 39 directional transmission links and 50 nondirectional transmission links. These values indicate a precision of 63% when including the direction of transmission, and 64% otherwise. Specificity remains very high in both cases (>99.9%). These values are summarized in Table 4

**Table 4:** Performance measures for the second simulation study

Performance measure	Original	Extra obs	Noisy 5	Noisy 10	Noisy 20	No demes
Sensitivity (dir.)	42%	55%	44%	41%	38%	34%
Sensitivity (non-dir.)	57%	69%	58%	56%	57%	54%
Specificity (dir.)	>99.9%	>99.9%	>99.9%	>99.9%	>99.9%	>99.9%
Specificity (non-dir.)	>99.9%	>99.9%	>99.9%	>99.9%	>99.9%	>99.9%
Precision (dir.)	63%	69%	64%	62%	58%	53%
Precision (non-dir.)	64%	73%	63%	65%	65%	63%

We evaluate the sensitivity, specificity, and precision for both directional transmission links (dir.) and nondirectional transmission links (non-dir.). We show results for the original simulated dataset (Original), using five samples per host (Extra obs), mislabeling 5%, 10%, and 20% of host demes (Noisy 5, Noisy 10, Noisy 20), and discarding deme information completely (No demes).

When undertaking inference without deme data, we assume that all hosts belong to the same deme, giving single *R* and *π* parameters, and removing all *ρ* parameters. The posterior means and credible intervals are presented in the “No demes” column of Table 3, with the single *R* and *π* parameters being assigned to $R_{1}$ and $\pi_{1}$, respectively. The estimates of *R* and *π* lie between the pairs of values $R_{1},R_{2}$ and $\pi_{1}$, $\pi_{2}$ used in the simulation. The true values of *κ* and *λ* remain in the 95% credible intervals, but the estimate of *λ* has slightly increased. The performance measures are summarized in Table 4 under the “No demes” column. Using the deme data improve the sensitivity when establishing both directional and nondirectional transmission links, as well as the precision for directional transmission links. The precision for nondirectional transmission links is similar.

**Fig. 4.**
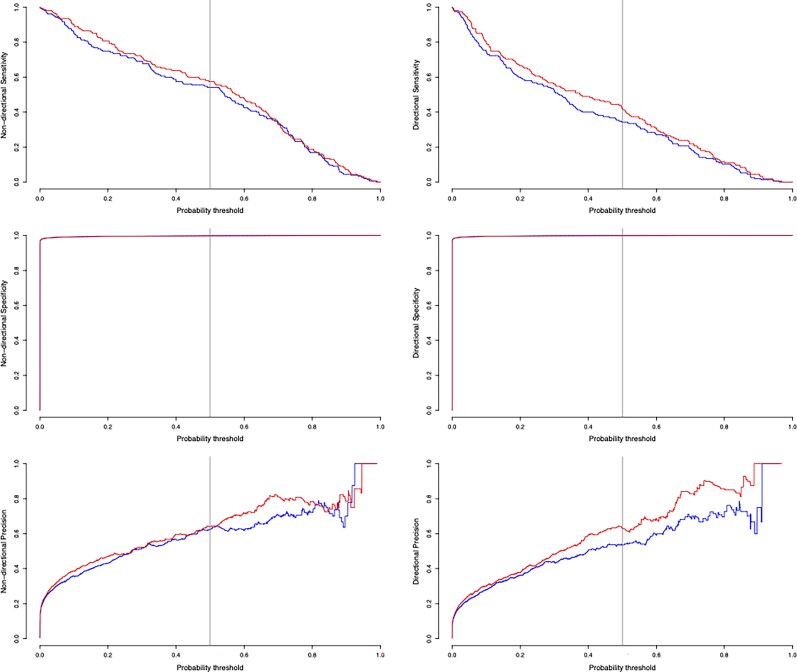
Performance measures for the second simulation study vs the probability threshold used to identify transmission links. Top: Sensitivity, middle: specificity, bottom: precision. On the left, we show nondirectional transmission links, and on the right we show directional transmission links. The red curves indicate the performance when using deme data, and the blue curves indicate performance when ignoring deme data. The vertical line at 0.5 shows the probability threshold used in our analysis.

We compare the performance measures across different threshold values in Fig. 4. There is an improvement in both sensitivity and precision when using the deme information. This is most notable when identifying directional transmission links.

We repeat the analysis after assigning 5%, 10%, and 20% of the observed hosts to the incorrect deme. We compare parameter estimates in Table 3 and performance measures in Table 4, under the “Noisy 5,” “Noisy 10,” and “Noisy 20” columns, respectively. As more errors are added, the separation between low transmission/poorly surveyed individuals and high transmission/well surveyed individuals becomes less clear, and so the estimated values of $R_{1}$, $R_{2}$, $\pi_{1}$, and $\pi_{2}$ become increasingly biased. The estimates of $\rho_{1}$ and $\rho_{2}$ decrease as more errors are added, as in the first simulation study. With 5% and 10% errors, the performance measures are broadly similar to the original analysis. With 20% errors we observe a decline in sensitivity and precision when establishing direct transmission links.

We repeat the analysis with five samples per host, comparing parameter estimations in Table 3 and performance measures in Table 4 under the “Extra obs” column. The estimates of *κ* and *λ* are again more strongly constrained. We also observe a strong improvement in both sensitivity and precision in comparison to the original analysis.

### Benchmarking Using Multiple Simulations

We undertake 50 simulation studies across a range of parameter values, similar in design to the second simulation study. The parameter sets are sampled from an orthogonal array Latin hypercube using the l hs R package. In each case, we simulate an outbreak with 250 observed hosts across two demes, with each observed host being sampled once. Each deme has separate *R* values sampled between 1 and 6, separate *π* values sampled between 0.1 and 1, and separate *ρ* values sampled between 0.5 and 0.9. Smaller values of *ρ* are not considered, as if *ρ* is high in one deme and low in the other this will lead to samples being dominated by one deme. In such cases, we would expect to obtain poor parameter estimates for the less sampled deme, as with few data points the posterior distributions would strongly resemble the prior distributions. The remaining parameters are the same for both demes, namely both *κ* and *λ* are sampled between 0 and 1. For each simulated dataset, we estimate the eight parameters used in the simulation.

**Fig. 5.**
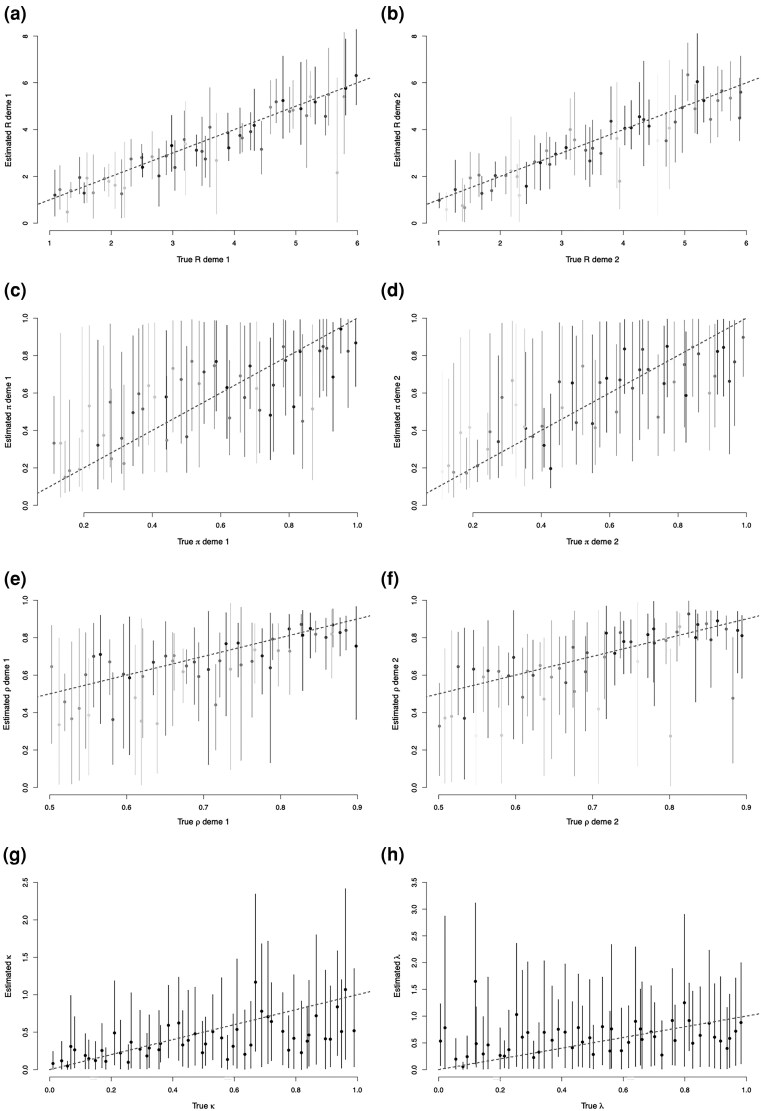
Benchmarking results for the parameters *R* of deme 1 a), *R* of deme 2 b), *π* of deme 1 c), *π* of deme 2 d), *ρ* of deme 1 e), *ρ* of deme 2 f), *κ* g), and *λ* h). Each *x* axis represents the true value used in the simulation, and each *y* axis represents inferred values. Posterior mean values are shown by dots, and 95% credible intervals are shown by vertical lines. The dashed line has slope 1 and intercept 0, showing where true parameter values equal estimated parameter values. Credible intervals that cross this line capture the true parameter values. In a–f), the shade indicates the proportion of sampled hosts in the associated deme (darker implying a greater proportion in the deme).

The marginal posterior credible intervals are shown in Fig. 5 and compared with the correct values of the parameters used in the simulations. In general, we are able to recover the parameter values used in each simulation, but there is considerable uncertainty on some of the parameters, as can be seen by the wide credible intervals in Fig. 5. Where the parameter estimates exhibit large uncertainty, the choice of prior distribution can be strongly influential. For example, we use a uniform prior distribution for each *π* parameter (prior mean 0.5), which can lead to the posterior means overestimating low values of *π*, and underestimating high estimates of *π*. We use exponential prior distributions on *κ* and *λ*, which concentrates prior mass on low values. Estimates for *κ* and *λ* can be significantly improved by adding more samples per host. In supplementary material Fig. S1, Supplementary Material online, we show the results of the benchmarking study when using five samples per host, and we are able to recover both *κ* and *λ* effectively.

Posterior uncertainty seems particular high for parameters of a deme with a small number of representative samples, as shown by the more lightly shaded bars being longer than the more darkly shaded bars in Fig. 5a–f. The parameter *ρ* in particular tends to be inferred more precisely when its true value is high (Fig. 5e–f). Formally, the correlation between the number of samples in the deme and the posterior variance lies between −0.51 and −0.25 for each parameter. These correlations are significant according to a one-sided correlation *t*-test at a 5% significance level.

For many of the simulations we also find large correlations (as high as 0.8) between *π* and *ρ* for opposing demes. That is, we can struggle to identify whether transmissions to a deme are rare and the deme is well-sampled (high $\rho_{i}$, high $\pi_{j}$, i≠j), or transmissions to a deme are common and the deme is poorly sampled (low $\rho_{i}$, low $\pi_{j}$, i≠j). As a demonstration, in supplementary material Fig. S2, Supplementary Material online, we plot the joint posterior distributions between every *π*, *ρ* pair in the first simulation. These high correlations contribute to the high posterior variances observed in the marginal posterior distributions. Additionally, since we do not consider values of *ρ* <0.5, our estimates do not include many small values of *π*.

We evaluate the performance measures across the 50 simulated datasets, using a posterior probability threshold of 0.5 to establish transmission links. We find a mean [range] directional sensitivity of 14% [4%, 33%], and nondirectional sensitivity 24% [6%, 48%]. These relatively low sensitivities are typical of datasets with one sample per observed host, and data sets with a relaxed bottleneck (Carson et al. 2024). We obtain specificities of >99.9% in all cases. The mean [range] directional precision is 61% [29%, 88%], and nondirectional precision 64% [33%, 88%]. The most influential parameter for the performance measures is the initial pathogen population size *κ*, which has an approximate correlation (averaging over directional and nondirectional measures) of −0.65 with sensitivity, 0.3 with specificity, and −0.25 with precision. A complete transmission bottleneck (κ=0) restricts the number of transmission trees that are compatible with a given phylogenetic tree, leading to high certainty when establishing transmission links. With a high *κ* there are many more plausible transmission trees, leading to lower posterior probability estimates for many transmission links, and hence lower numbers of both true and false positives. For sensitivity, *λ* is also influential (correlation −0.3), as a high *λ* increases the probability of transmissions including multiple lineages, and hence increasing the uncertainty of transmission links. For specificity, the *π* parameters are influential (correlation −0.45 for $\pi_{1}$ and −0.3 for $\pi_{2}$). Low *π* values increase the probability that there are intermediary transmission links between observed individuals, lowering the posterior probability of direct transmission links. The higher correlation with $\pi_{1}$ compared with $\pi_{2}$ may arise from earlier samples being predominantly from deme 1. Our root host always starts off in deme 1, and so earlier samples are more likely to arise from deme 1.

We obtain the sensitivity (recall), specificity, and precision for each simulation under a series of different posterior probability thresholds between 0 and 1. Averaging over the 50 simulated datasets, we present the resulting receiver operating characteristic (ROC) and precision-recall (PR) curves in supplementary material Fig. S3, Supplementary Material online for both directional and nondirectional transmission links. The area under the curve is 0.99 for both ROC curves, 0.33 for the PR curve of directional transmission links, and 0.44 for the PR curve of nondirectional transmission links.

### Application to an Outbreak of Tuberculosis in Argentina

A multidrug-resistant *Mycobacterium tuberculosis* outbreak in Argentina has been described and studied in detail using genomic epidemiology (Eldholm et al. 2015). A total of 252 genomes were sequenced, with collection dates ranging between October 1996 and December 2009. One hundred fifty-three of the genomes originated from HIV positive individuals, whereas the remaining 99 genomes where sampled from HIV negative individuals. A dated phylogeny was previously reconstructed using BEAST (Drummond et al. 2012), with the root of this tree being estimated to have existed around 1970 (Eldholm et al. 2015). This dated phylogeny is the starting point of our analysis and reproduced in supplementary material Fig. S4, Supplementary Material online, with leaves colored according to the HIV status of the hosts. The role of HIV coinfection in the transmission of this tuberculosis outbreak has been previously investigated and found to be not statistically significant (Eldholm et al. 2016). However, this analysis was based on a rough reconstruction of transmission events, with a posteriori testing of the effect of HIV status, limiting its statistical power (Eldholm et al. 2016). It is therefore interesting to reanalyze this dataset with the new methodology presented here, considering two demes for the HIV positive and negative individuals. The sampling window was set from 1st October 1996 to 1st December 2009 to include all samples and reflect the original sampling collection methodology (Eldholm et al. 2015). We used the same generation time and sampling time distributions as was used in previous analyses of tuberculosis outbreaks (Didelot et al. 2017; Séraphin et al. 2018; Sobkowiak et al. 2023; Chitwood et al. 2025).

**Fig. 6.**
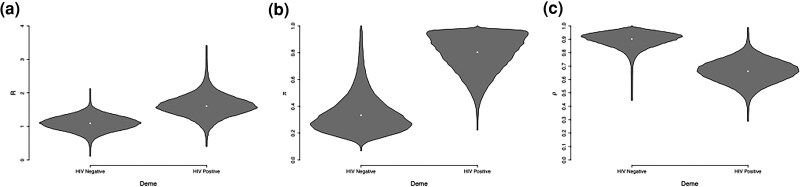
Parameter estimates in the tuberculosis analysis: the reproduction number *R* a), sampling fraction *π* b), and probability to remain in a deme *ρ* c) are shown for both the HIV negative (left) and HIV positive (right) demes.

The means (95% credible intervals) of the parameters of the within-host population size function are κ:5.38(2.82,8.79) and λ:0.63(0.04,1.69). The initial pathogen population size *κ* is large compared with the per-year linear growth rate *λ*, suggesting a relaxed transmission bottleneck (Carson et al. 2024). Figure 6 shows the posterior distribution for the parameters specific to both demes. The reproduction number *R* for the HIV negative and HIV positive demes are 1.10(0.65,1.52) and 1.63(0.86,2.32), respectively (Fig. 6a). The probability that the HIV positive deme has a greater reproduction number than the HIV negative deme is 0.86. The sampling probability *π* for the HIV negative and HIV positive demes are 0.32(0.13,0.70) and 0.79(0.46,0.99), respectively (Fig. 6b). The probability that the HIV positive deme has a greater sampling probability than the HIV negative deme is 0.98. These two comparisons suggest that HIV positive individuals both have a greater reproduction number (as indicated by the larger *R* estimate), and are more likely to be observed (as indicated by the larger *π* estimate). This may be expected from the fact that HIV coinfection accelerates the transition from latent to active tuberculosis (Bruchfeld et al. 2015).

**Fig. 7.**
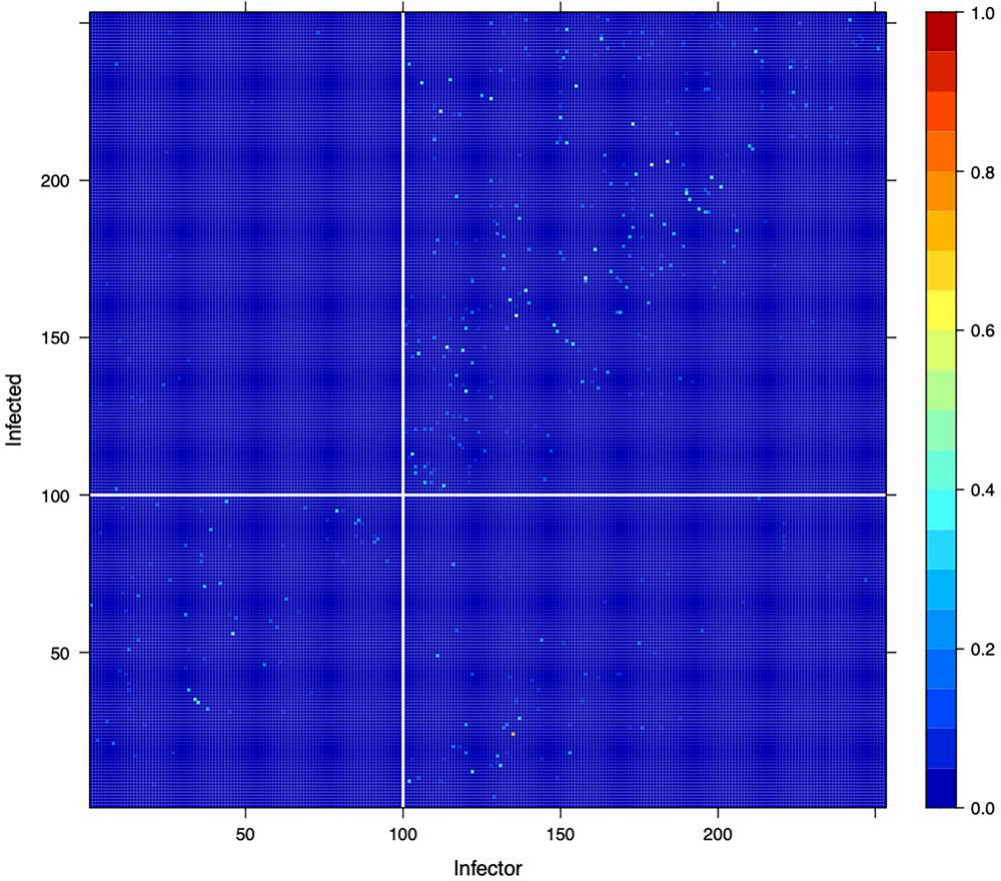
Posterior transmission probabilities in the tuberculosis analysis. The *x* axis indicates the infector, and the *y* axis indicates the infected. The shading in each cell indicates the posterior probability that the infector transmitted directly to the infected. We have left gaps to separate individuals who are HIV negative (left/bottom) and HIV positive (right/top).

Figure 7 shows the posterior probabilities of direct transmission from any individual to any other. It is visually clear that infector/infected pairs tend to have the same HIV status. This is confirmed by the estimate of the probabilities that the pathogen remains in the same deme at transmission, which are 0.90(0.77,0.98) and 0.66(0.48,0.82), respectively, for the HIV negative and HIV positive demes (Fig. 6c). Therefore, HIV negative hosts are highly likely to transmit to other HIV negative hosts. HIV positive hosts are also more likely to transmit to other HIV positive hosts, but to a lesser extent. This may be in part due to the HIV negative population being larger than the HIV positive population. The association between tuberculosis transmission and HIV status is likely the result of the underlying socioeconomic structure of the human population, which underpins the contact networks of both diseases (Mills et al. 2011).

### Application to an Outbreak of Avian Flu H7N7 Outbreak in the Netherlands

An outbreak of avian influenza H7N7 occurred in the Netherlands during 2003, infecting 255 Dutch farms in <3 months, and leading to drastic control measures including the culling of 30 million birds (Stegeman et al. 2004). Genetic data are available from GISAID (Shu and McCauley 2017) from 227 farms, for genes HA, NA, and PB2 which were concatenated. Most sequences are from the Gelderland (G) area (n=186) with smaller numbers from the Limburg (L) area (n=33), Central (C) area (n=7), and Southwest (S) area (n=1). The phylogeography of this outbreak has been described before in a number of studies (Bataille et al. 2011; Ypma et al. 2012, 2013; Hall et al. 2015; Klinkenberg et al. 2017). We built a dated tree using BEAST2 (Bouckaert et al. 2019) which is shown in supplementary material Fig. S5, Supplementary Material online with leaves colored by location. We used the same generation time and sampling time distributions as in a recent study of this outbreak (Klinkenberg et al. 2017). The sampling window was set from the 50th to the 125th day from the root of the dated tree, which included all samples (supplementary material Fig. S5, Supplementary Material online).

**Fig. 8.**
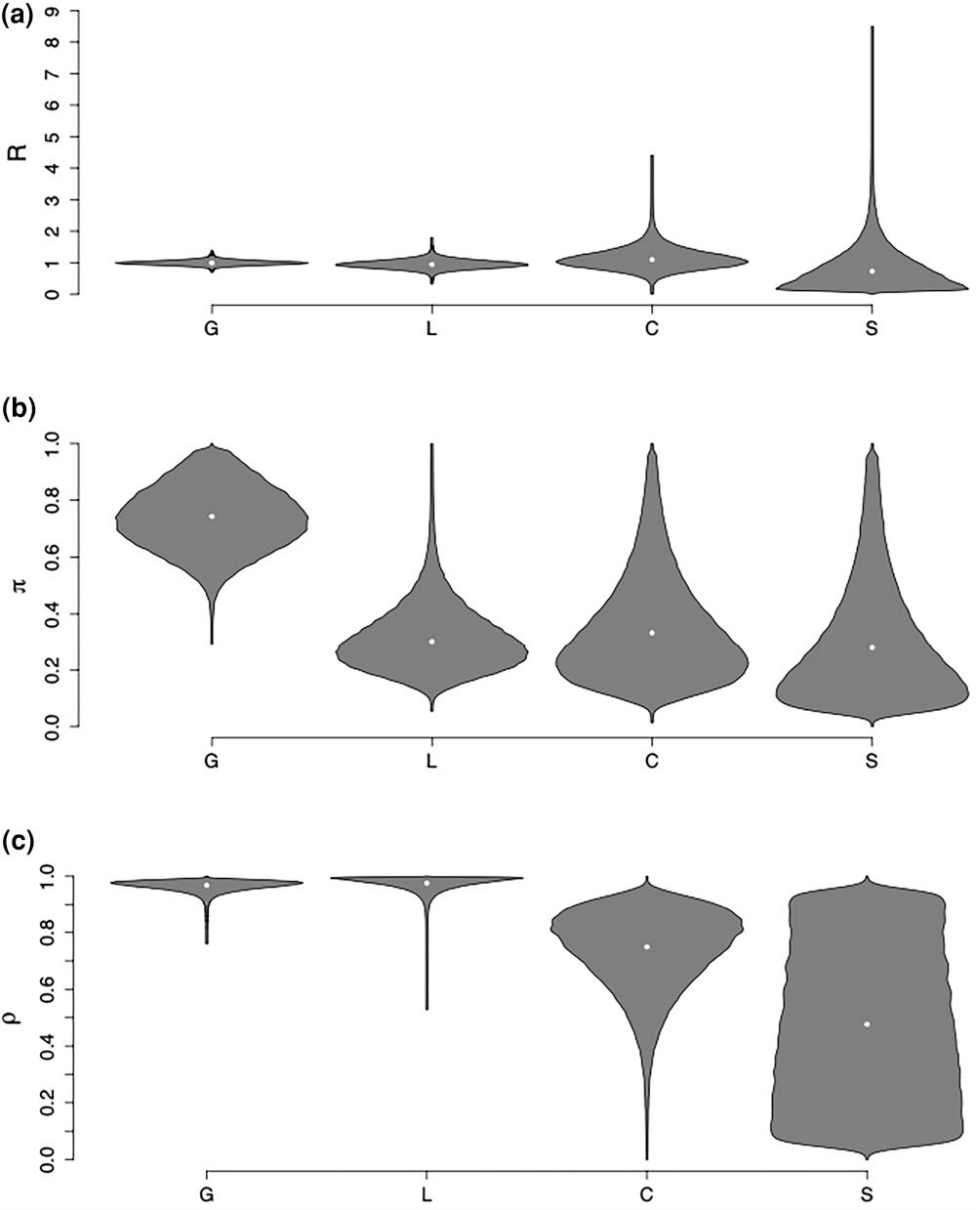
Parameter estimates in the H7N7 analysis: the reproduction number *R* a), sampling proportion *π* b), and probability to remain in a deme *ρ* c) are shown for each of the four locations.

The means (95% credible intervals) of the parameters of the within-host population size function are κ:5.38(2.23,9.60) and λ:3.55(1.24,6.28). Figure 8 shows the posterior distribution for the parameters specific to the four locations. The per-location reproduction numbers are 1.00(0.86,1.16), 0.98(0.71,1.25), 1.13(0.54,1.93), and 0.74(0.02,2.37) for regions G, L, C, and S, respectively. These reproduction numbers are close to 1, with uncertainty increasing as the sample numbers decrease (Fig. 8a). In location S, we approximately recover the prior exponential with mean 1, as would be expected given that there was only a single representative of this location. The sampling probabilities *π* are 0.71(0.36,0.97), 0.28(0.10,0.55), 0.33(0.07,0.82), and 0.27(0.02,0.83) for regions G, L, C, and S, respectively. Location G is best sampled, which makes sense given that it has the largest number of sampled cases (Fig. 8b). The remaining demes are likely less well sampled, although there was high uncertainty due to the small sample numbers. Our analysis was conducted on 227 farms for which genetic data was available, whereas during the outbreak 255 farms were confirmed to be infected (Stegeman et al. 2004; Bataille et al. 2011), suggesting an upper bound of the sampling fraction of 0.89. Our estimates are compatible with this, and further suggest that some infection went undetected as would be expected from the large-scale culling that took place at farms even in the absence of detection (Stegeman et al. 2004).

**Fig. 9.**
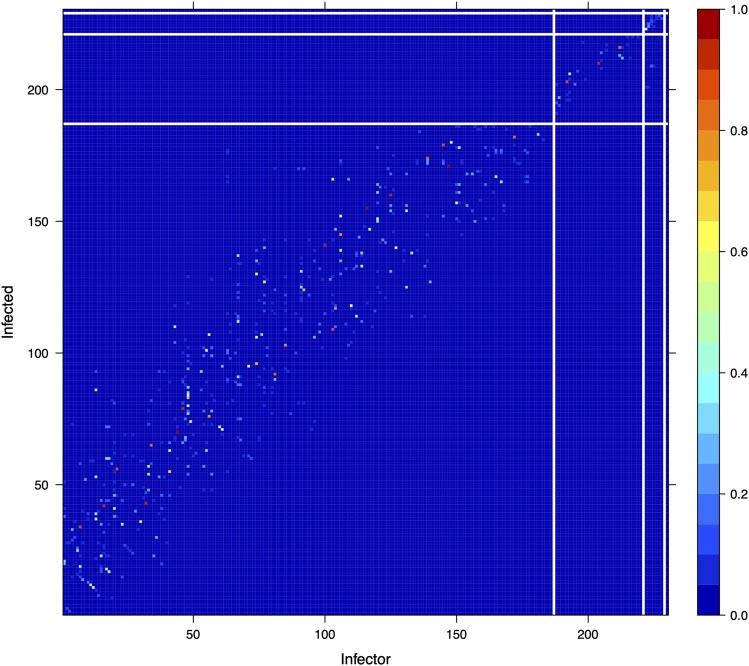
Posterior transmission probabilities in the H7N7 analysis. The *x* axis indicates the infector and the *y* axis indicates the infected. The shading in each cell indicates the posterior probability that the infector transmitted directly to the infected. We have left gaps to separate hosts at the four locations.

Figure 9 shows the posterior probabilities of direct transmission from any farm to any other, with a clear tendency for farms to infect other farms from the same region. This is confirmed by the parameters *ρ* representing the probability that the pathogen infects in the same location, which are estimated to be 0.97(0.92,0.99), 0.98(0.92,1.00), 0.75(0.4,0.95), and 0.48(0.02,0.97) for regions G, L, C, and S, respectively (Fig. 8c). Note that we assume that transmissions to different locations are evenly distributed across the other locations. Farms in locations G and L almost always transmit to offspring in the same deme. The probability that a farm in location C infects another farms in location C also seems high, but is more uncertain due to the small number of samples. For location S represented by only a single farm, we approximately recover the prior uniform between 0 and 1.

## Discussion

Combining genomic and epidemiological data to reconstruct the transmission events within an infectious disease epidemic is an idea that was formulated over a decade ago, when the first methods to use genomic data for outbreak reconstruction were proposed (Ypma et al. 2012; Jombart et al. 2014; Didelot et al. 2014; Hall et al. 2015). It is, however, difficult to use epidemiological data when considering partially sampled or ongoing outbreaks (Mollentze et al. 2014; Didelot et al. 2017), since unsampled cases do not have any associated epidemiological data. A naive approach to this issue quickly becomes intractable as larger numbers of unsampled cases need to be considered. Instead, we presented a new dynamic programming algorithm that can efficiently resolve this problem. We implemented this new methodology by extending the TransPhylo framework which can be applied to partially sampled outbreaks (Didelot et al. 2017, 2021), even when multiple genomes per host are provided or when the transmission bottleneck is not complete (Carson et al. 2024).

We used simulations to show that the combined approach has improved statistical power to infer the correct transmission events compared with the previous approach based on genomic data only. We also showed that the parameters governing the epidemiological data can be inferred with accuracy that increases with the amount of data available for analysis. We applied our new algorithm to two widely different real datasets to showcase the range of scenarios in which it can be useful. First, we analyzed data from a tuberculosis outbreak in Argentina (Eldholm et al. 2015) to investigate the role of HIV coinfection on the spread of the bacterial causative agent *M. tuberculosis*. Second, we analyzed data from the avian influenza H7N7 epidemic that hit the Netherlands in 2003 (Stegeman et al. 2004), to infer the parameters involved in the spatial spread of this virus from farm to farm. Such real-life applications pose additional challenges compared with applications to simulated data. Real data can contain mistakes and we have shown that our approach is relatively robust to this. More importantly, it is important to consider whether the infectious disease outbreak under study meets the assumptions of our model, summarized below.

The methodological framework we developed makes few assumptions, and we therefore envisage that it can be useful in a wide range of situations. The epidemiological model at the heart of TransPhylo is a flexible branching process (Didelot et al. 2017) based on offspring distribution and generation time distribution whose parameters can be set to appropriately model many infectious diseases transmitted directly from host to host (Wallinga and Teunis 2004; Grassly and Fraser 2008; Cori et al. 2013). An example of application concerns the inference of the different reproduction numbers for different components of the population. This can help determine their relative contribution to the overall disease burden, and therefore inform how to target public heath policies for maximum effect (Fraser et al. 2004; Grassly and Fraser 2008; Hollingsworth 2009). Another application likely to be useful is to estimate the sampling proportions for different components of the population, which can reveal if sampling is currently biased and how it could be improved (Magnani et al. 2005; Brooks-Pollock et al. 2021; Layan et al. 2023).

There are, however, some limitations to the methodology we presented. First, we only considered discrete epidemiological data. Continuous variables can always be discretized to circumvent this limitation, but doing so may lose some information and requires to define potentially arbitrary discrete classes. This situation is analogous to the almost ubiquitous use of discrete locations in phylogeography (Lemey et al. 2009; De Maio et al. 2015; Baele et al. 2017). Second, we make the assumption that hosts with deme data have been assigned to the correct deme. Our simulation studies show that performance can degrade if the deme data contain errors. It is possible to extend the dynamic programming algorithm to include such observation errors: instead of assigning probability 1 to a sampled host’s deme, we could instead assign probability p<1, with probability 1−p being split across the remaining demes. The new parameter *p*, reflecting the proportion of correctly assigned demes, would need to be inferred. Third, in our applications, we assume that transmission outside of a deme is evenly distributed across the other demes. We make this choice in order to restrict the number of parameters to be linear in the number of demes instead of quadratic, but recognize that it will not be appropriate for every situation. The dynamic programming algorithm allows for this assumption to be relaxed, but a more complex MCMC algorithm would be required to efficiently estimate the additional model parameters. Other parametrizations could also be useful in some situations, for example having a parameter for the expected flow into each deme which would be linear with the number of demes. Fourth, we see through our simulation studies and applications that an unbalanced population structure leads to larger uncertainties in the parameter estimates for the less sampled demes. Furthermore, as we are taking a Bayesian approach, parameter estimates for the less sampled demes will be more strongly influenced by the choice of prior distribution. Some care must therefore be taken when interpreting the results of such analyses. Note that any extension that adds parameters to the model will likely increase uncertainty in the posterior estimates.

Each analysis is based on a dated phylogeny that needs to be correctly precomputed. This requires consideration of how such a tree is computed, under which prior model if a Bayesian method is used, and to what extent a single point estimate can be used without quantification of uncertainty. These questions arise for all of the many recently developed phylodynamic methods that take a dated tree as input (Didelot and Parkhill 2022). However, this step-by-step approach is necessary to be able to analyze state-of-the-art large genomic datasets. Finally, it should be noted that our methodology has a nonnegligible computational cost. For example, the largest analysis we performed, on the H7N7 dataset, took several days to achieve acceptable MCMC convergence and mixing properties. However, our algorithm currently runs only on a single CPU core, whereas most standard desktop and laptop computers have 8 to 16 cores, with many more cores available on servers dedicated to computer-intensive tasks. Future work should therefore seek to exploit multiple cores to reduce the overall runtime, for example by following recent progress in parallel MCMC algorithms (Schwedes and Calderhead 2021; Syed et al. 2022; Glatt-Holtz et al. 2024).

## Methods

### Case Where All Demes Have the Same Offspring Distribution and Sampling Probabilities

Let us start with the simpler case where all demes are assumed to have the same offspring distribution and sampling probabilities. In this case, most of the calculations in TransPhylo (Didelot et al. 2017; Carson et al. 2024) remain unchanged, we simply include an additional likelihood term obtained by using an efficient dynamic programming algorithm similar to Felsenstein’s tree-pruning algorithm (Felsenstein 1973, 1981). This is necessary to integrate over demes for unsampled individuals that form part of the transmission tree and for sampled individuals with missing deme data.

Let hosts be labeled 1,…,N, and define the deme of host *n* by $s^{n}\in \{1,\ldots ,S\}$. Let $P_{ij}$ be the probability that an offspring of a host in deme *i* is in deme *j*. Finally, let $L_{s}^{n}$ be the likelihood from the deme data of host *n* and their descendants, conditional on host *n* being in deme *s*. The algorithm is initialized at the leaf nodes, which in this case are hosts with no offspring in the transmission tree, noting that all such hosts must have been sampled in TransPhylo. If the deme of such a host is known, then $L_{s^{n}}^{n}=1$ at the hosts deme $s^{n}$, and $L_{s}^{n}=0$ for $s\neq s^{n}$ (all other demes). If the deme of the host is unknown, then $L_{s}^{n}=1$ for all possible demes *s*.

The algorithm then proceeds backward in time to evaluate the conditional likelihoods of the internal nodes (hosts with offspring). Let $H^{n}$ denote the set of offspring of Host *n*. If Host *n* has a known deme $s^{n}$ then


(1) $$L_{s^{n}}^{n}=\prod_{\;j\in H^{n}}\sum_{s^{j}=1}^{S}P_{s^{n}s^{j}}L_{s^{j}}^{j},$$

and $L_{s}^{n}=0$ for $s\neq s^{n}$. If on the other hand Host *n* has no known deme then


(2) $$L_{s}^{n}=\prod_{\;j\in H^{n}}\sum_{s^{j}=1}^{S}P_{ss^{j}}L_{s^{j}}^{j},$$

for all possible values of *s*.

The algorithm terminates at the root host, assumed here to be n=1. The overall likelihood of the demes on the transmission tree is given by


(3) $$L=\sum_{s=1}^{S}\varsigma_{s}L_{s}^{1},$$

where $\varsigma_{s}$ is the prior probability of the root host being in deme *s*. The overall cost of the algorithm is expected to scale $O(NS^{2})$, as we need to evaluate the likelihood for every deme combination across every individual and their infector.

### Illustrative Example

An example of the dynamic programming algorithm is shown in supplementary material Fig. S6a, Supplementary Material online. The target transmission tree contains 10 hosts and we assume that there are three possible demes. The transition probability between the three demes is given by


(4) $$P=\left(\begin{array}{ccc} 0.8 & 0.1 & 0.1\\ 0.1 & 0.8 & 0.1\\ 0.1 & 0.1 & 0.8 \end{array}\right),$$

meaning that a host has probability 0.8 of an offspring having the same deme as its infector, and a probability 0.1 of an offspring being in either of the other two demes. We know that Host 5 is in deme 1, Hosts 3 and 7 are in deme 2, and Host 10 is in deme 3. Hosts 2, 4, and 9 are sampled hosts, but their deme is missing. Hosts 1, 6, and 8 are unsampled hosts.

Each host has an associated vector for the conditional likelihood at the three demes. Working backward in time, Host 10 is known to be in deme 3 and is a leaf, and so:


(5) $$L^{10}=\left(\begin{array}{c} 0\\ 0\\ 1 \end{array}\right).$$

Host 9 is a leaf, but does not have a known deme, so that:


(6) $$L^{9}=\left(\begin{array}{c} 1\\ 1\\ 1 \end{array}\right).$$

Host 8 is an unsampled individual, whose only offspring is Host 10:


(7) $$L^{8}=\left(\begin{array}{c} 0.8\cdot 0+0.1\cdot 0+0.1\cdot 1\\ 0.1\cdot 0+0.8\cdot 0+0.1\cdot 1\\ 0.1\cdot 0+0.1\cdot 0+0.8\cdot 1 \end{array}\right)=\left(\begin{array}{c} 0.1\\ 0.1\\ 0.8 \end{array}\right).$$

Host 7 is another leaf with deme 2:


(8) $$L^{7}=\left(\begin{array}{c} 0\\ 1\\ 0 \end{array}\right).$$

Host 6 is an unsampled individual, whose only offspring is Host 9:


(9) $$L^{6}=\left(\begin{array}{c} 0.8\cdot 1+0.1\cdot 1+0.1\cdot 1\\ 0.1\cdot 1+0.8\cdot 1+0.1\cdot 1\\ 0.1\cdot 1+0.1\cdot 1+0.8\cdot 1 \end{array}\right)=\left(\begin{array}{c} 1\\ 1\\ 1 \end{array}\right).$$

Host 5 is a leaf with deme 1:


(10) $$L^{5}=\left(\begin{array}{c} 1\\ 0\\ 0 \end{array}\right).$$

Host 4 is a leaf with no deme data:


(11) $$L^{4}=\left(\begin{array}{c} 1\\ 1\\ 1 \end{array}\right).$$

Host 3 has three offspring: Hosts 4, 8, and 7. Additionally, Host 3 is in deme 2, and so we only calculate the second element of the vector:


(12) $$L^{3}=\left(\begin{array}{c} 0.0\\ \left(0.1\cdot 1+0.8\cdot 1+0.1\cdot 1)(0.1\cdot 0+0.8\cdot 1+0.1\cdot 0)(0.1\cdot 0.1+0.8\cdot 0.1+0.1\cdot 0.8\right)\\ 0.0 \end{array}\right)=\left(\begin{array}{c} 0.000\\ 0.136\\ 0.000 \end{array}\right).$$

Host 2 has two offspring: Hosts 5 and 6. Host 2 is sampled, but has no deme data, and so all elements of the vector are evaluated:


(13) $$L^{2}=\left(\begin{array}{c} \left(0.8\cdot 1+0.1\cdot 0+0.1\cdot 0)(0.8\cdot 1+0.1\cdot 1+0.1\cdot 1\right)\\ \left(0.1\cdot 1+0.8\cdot 0+0.1\cdot 0)(0.1\cdot 1+0.8\cdot 1+0.1\cdot 1\right)\\ \left(0.1\cdot 1+0.1\cdot 0+0.8\cdot 0)(0.1\cdot 1+0.1\cdot 1+0.8\cdot 1\right) \end{array}\right)=\left(\begin{array}{c} 0.8\\ 0.1\\ 0.1 \end{array}\right).$$

Finally, Host 1 has two offspring: Hosts 2 and 3:


(14) $$L^{1}=\left(\begin{array}{c} \left(0.8\cdot 0.8+0.1\cdot 0.1+0.1\cdot 0.1)(0.8\cdot 0.0+0.1\cdot 0.136+0.1\cdot 0.0\right)\\ \left(0.1\cdot 0.8+0.8\cdot 0.1+0.1\cdot 0.1)(0.1\cdot 0.0+0.8\cdot 0.136+0.1\cdot 0.0\right)\\ \left(0.1\cdot 0.8+0.1\cdot 0.1+0.8\cdot 0.1)(0.1\cdot 0.0+0.1\cdot 0.136+0.8\cdot 0.0\right) \end{array}\right)=\left(\begin{array}{c} 0.008976\\ 0.018496\\ 0.002312 \end{array}\right).$$

supplementary material Figure S6b, Supplementary Material online shows the transmission tree annotated with the conditional likelihoods calculated in the dynamic programming algorithm. If we assume that the prior for the deme of the root host is one-third for the three demes, then the likelihood of the deme is the mean of the values for Host 1. In this case L=0.009928. This is verified by brute force by calculating


(15) $$L=\sum_{s^{1}=1}^{S}\sum_{s^{2}=1}^{S}\sum_{s^{4}=1}^{S}\sum_{s^{6}=1}^{S}\sum_{s^{8}=1}^{S}\sum_{s^{9}=1}^{S}\pi_{s^{1}}P_{s^{1}s^{2}}P_{s^{1}s^{3}}P_{s^{2}s^{5}}P_{s^{2}s^{6}}P_{s^{3}s^{4}}P_{s^{3}s^{7}}P_{s^{3}s^{8}}P_{s^{6}s^{9}}P_{s^{8}s^{10}}.$$

Note that leaves with no deme data do not ultimately contribute to the likelihood, and can therefore be excluded.

### Case Where the Demes May Have Different Offspring Distributions and Sampling Probabilities

A useful extension would be to allow *R* and/or *π* to change based on deme. For now, let us assume that there are S=2 demes with offspring distribution $\alpha_{1}(k)$ and $\alpha_{2}(k)$. Host infected at time *t* are observed with probability $\pi_{1}$ and $\pi_{2}$ (leading to time-dependent probabilities $\zeta_{1}(t)$ and $\zeta_{2}(t)$). Unlike the previous case, the transmission tree likelihood and deme likelihood can not be calculated separately. To evaluate the combined likelihood, we start by calculating the exclusion probabilities as follows.

Define $\omega_{1}(t)$ as the exclusion probability of a host infected at time *t* in deme 1, and $\omega_{2}(t)$ as the exclusion probability of a host infected at time *t* in deme 2. Assuming that *T* is the cutoff time for observations $\omega_{1}(t)=\omega_{2}(t)=1$ for t≥T. We can then define the following recursive relationships.

The exclusion probability of an offspring from a host in deme 1 infected at time *t* is


(16) $${\bar{\omega }}_{1}(t)=\int_{0}^{\infty }(P_{11}\omega_{1}(t+\tau )+P_{12}\omega_{2}(t+\tau ))\gamma (\tau )\;d\tau ,$$

and for deme 2


(17) $${\bar{\omega }}_{2}(t)=\int_{0}^{\infty }(P_{21}\omega_{1}(t+\tau )+P_{22}\omega_{2}(t+\tau ))\gamma (\tau )\;d\tau .$$

The probability that all offspring from an individual in deme *i* infected at time *t* are excluded is


(18) $$\phi_{i}(t)=\sum_{k=0}^{\infty }\alpha (k)\bar{\omega_{i}}{\left(t\right)}^{k}.$$

The exclusion probability of an individual in deme *i* infected at time *t* is then


(19) $$\omega_{i}(t)=(1-\zeta_{i}(t))\phi_{i}(t).$$

That is, the probability of the host being unobserved and having no included offspring.

As established in Carson et al. (2024), the transmission tree likelihood contribution from an unsampled Host *n* is


(20) $$\frac{\left(1-\zeta (x^{n})\right)}{1-\omega (x^{n})}\sum_{k=d^{n}}^{\infty }\alpha (k)\left(\begin{array}{c} k\\ d^{n} \end{array}\right)\bar{\omega }(x^{n}{)}^{k-d^{n}}d^{n}!\prod_{\;j\in H^{n}}\left(1-\omega (x^{j}))\gamma (x^{j}-x^{n}\right),$$

where $x^{n}$ is the host’s infection time and $d^{n}$ is the number of included offspring. If Host *n* is sampled, the likelihood contribution is


(21) $$\frac{\pi \sigma (y^{n}-x^{n})}{1-\omega (x^{n})}\sum_{k=d^{n}}^{\infty }\alpha (k)\left(\begin{array}{c} k\\ d^{n} \end{array}\right)\bar{\omega }(x^{n}{)}^{k-d^{n}}d^{n}!\prod_{\;j\in H^{n}}\left(1-\omega (x^{j}))\gamma (x^{j}-x^{n}\right),$$

where $y^{n}$ is the host’s primary observation time and σ(τ) is the observation time distribution. Here, we define


(22) $$T_{s}^{n}=\frac{\left(1-\zeta_{s}(x^{n})\right)}{1-\omega_{s}(x^{n})}\sum_{k=d^{n}}^{\infty }\alpha_{s}(k)\left(\begin{array}{c} k\\ d^{n} \end{array}\right){\bar{\omega }}_{s}(x^{n}{)}^{k-d^{n}}d^{n}!$$

for an unobserved Host *n* in deme *s*, and


(23) $$T_{s}^{n}=\frac{\pi_{s}\sigma (y^{n}-x^{n})}{1-\omega_{s}(x^{n})}\sum_{k=d^{n}}^{\infty }\alpha_{s}(k)\left(\begin{array}{c} k\\ d^{n} \end{array}\right){\bar{\omega }}_{s}(x^{n}{)}^{k-d^{n}}d^{n}!$$

for an observed Host *n* in deme *s*. In addition, we define


(24) $$U_{ss^{j}}^{nj}=(1-\omega_{s^{j}}(x^{j}))\gamma (x^{j}-x^{n})P_{ss^{j}}$$

for $j\in H^{n}$ being the offspring of Host *n*, and $s^{j}$ being the deme of the offspring. Finally, define


(25) $$L_{s}^{n}=T_{s}^{n}$$

for leaf hosts, and


(26) $$L_{s}^{n}=T_{s}^{n}\prod_{\;j\in H^{n}}\sum_{s^{j}=1}^{S}U_{ss^{j}}^{nj}L_{s^{j}}^{j}$$

for hosts with offspring. The combined transmission tree and deme likelihood is then calculated using dynamic programming with this replacement definition of $L_{s}^{n}$.

### Illustrative Example

We return to the transmission tree presented in supplementary material Fig. S6a, Supplementary Material online. We include observation times as follows:

**Table:** 

Host	Deme	Infection time	Observation time	Offspring
1	–	0.0	–	2, 3
2	–	2.6	3.5	5, 6
3	2	3.2	5.3	4, 7, 8
4	–	5.1	6.9	–
5	1	5.2	8.5	–
6	–	5.5	–	9
7	2	6.3	7.1	–
8	–	7.1	–	10
9	–	8.9	11.0	–
10	3	9.8	11.6	–

We assume that the demes have basic reproduction number *R* equal to 2, 1.5, and 1, respectively, and sampling proportion *π* equal to 0.5, 0.7, and 0.9, respectively. We again set *P* as in Equation (4). Both the generation time distribution and observation time distribution are Gamma distributed with shape 2 and scale 1. The resulting exclusion probabilities are shown in supplementary material Fig. S7, Supplementary Material online.

The resulting conditional likelihoods are as follows:

**Table:** 

Host	Deme 1	Deme 2	Deme 3
1	$1.35\times 10^{-19}$	$1.24\times 10^{-19}$	$2.60\times 10^{-21}$
2	$9.71\times 10^{-8}$	$1.07\times 10^{-8}$	$4.86\times 10^{-9}$
3	0	$3.08\times 10^{-9}$	0
4	$5.67\times 10^{-2}$	$9.84\times 10^{-2}$	$1.53\times 10^{-1}$
5	$2.34\times 10^{-2}$	0	0
6	$2.41\times 10^{-3}$	$1.72\times 10^{-3}$	$6.20\times 10^{-4}$
7	0	$1.30\times 10^{-1}$	0
8	$1.40\times 10^{-3}$	$7.30\times 10^{-4}$	$1.80\times 10^{-3}$
9	$1.25\times 10^{-1}$	$1.68\times 10^{-1}$	$2.14\times 10^{-1}$
10	0	0	$3.61\times 10^{-1}$

The overall likelihood is $L=8.69\times 10^{-20}$, which again is confirmed by using a brute force calculation.

As a further check, we recalculate the likelihood under R=(2,2,2) and π=(0.8,0.8,0.8). As the demes now have the same offspring distribution and sampling probabilities, we should obtain the same likelihood by taking the product of the transmission tree and deme likelihoods, as in the case where all demes have the same offspring distribution and sampling probabilities. We find that both approaches do indeed return the same likelihood.

### Implementation

We implemented the methods above into a new R package called TransPhylo2, which extends TransPhyloMulti (Carson et al. 2024) and therefore inherits the same advantages over the previous implementation of TransPhylo (Didelot et al. 2017) in terms of allowing multiple samples per host and relaxing the assumption of a complete transmission bottleneck. TransPhylo2 is available at https://github.com/DrJCarson/TransPhylo2. This repository also contains all the code and data needed to reproduce all results shown in this paper. The R package ape was used to store, manipulate, and visualize phylogenetic trees (Paradis and Schliep 2019).

## Supplementary Material

msaf083_Supplementary_Data

## Data Availability

All the data and code needed to reproduce all results are available online at https://github.com/DrJCarson/TransPhylo2.
