# Supplementary material for: Incorporating Epidemiological Data into the Genomic Analysis of Partially Sampled Infectious Disease Outbreaks
Source: Mol Biol Evol. 2025 Apr 21;42(4):msaf083. doi: 10.1093/molbev/msaf083 (PMC12010114; doi:10.1093/molbev/msaf083)
Supplement: msaf083_Supplementary_Data [file msaf083_supplementary_data.pdf]

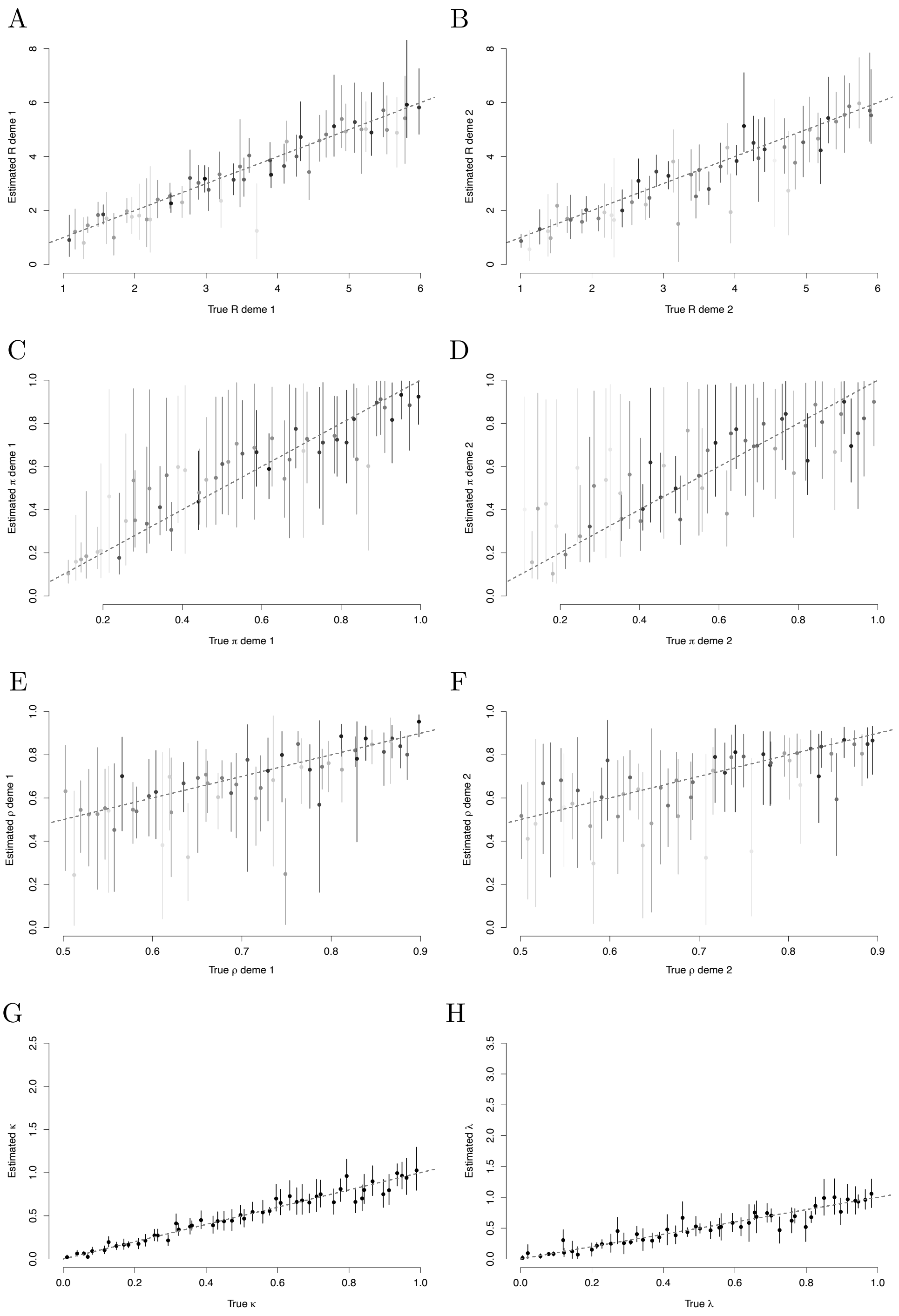

Figure S1: Benchmarking results with five samples per observed host for the parameters R of deme 1 (A), R of deme 2 (B), π of deme 1 (C), π of deme 2 (D), ρ of deme 1 (E), ρ of deme 2 (F), κ (G) and λ (H). Each x-axis represents the true value used in the simulation, and each y-axis represents inferred values. Posterior mean values are shown by dots, and 95% credible intervals are shown by vertical lines. The dashed line has slope 1 and intercept 0, showing where true parameter values equal estimated parameter values. Credible intervals that cross this line capture the true parameter values. In A-F the shade indicates the proportion of sampled hosts in the associated deme (darker implying a greater proportion in the deme). We maintain the same axis scales as Figure 5 for easy comparison.

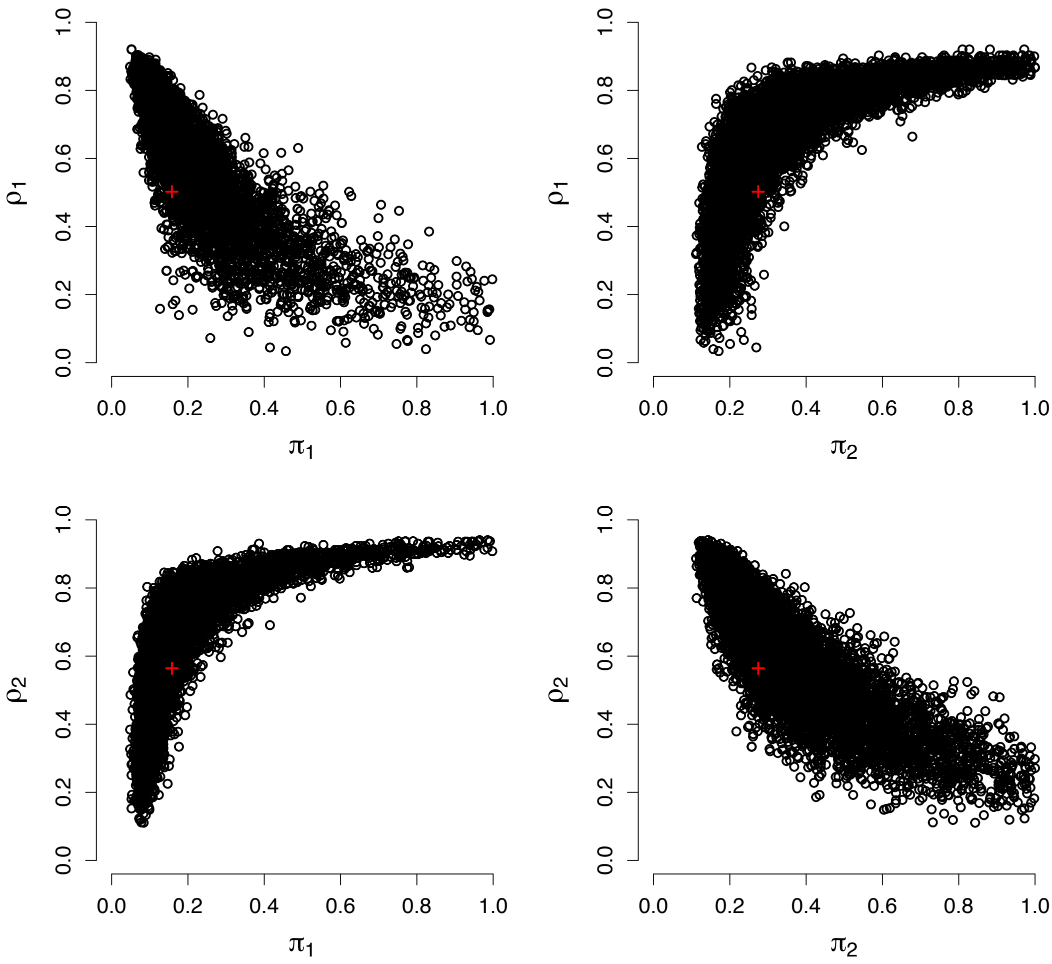

Figure S2: Posterior samples for the first simulated dataset in the benchmarking study. We show the joint posterior samples for π and ρ between each deme. The red mark indicates the parameter values used for the simulation.

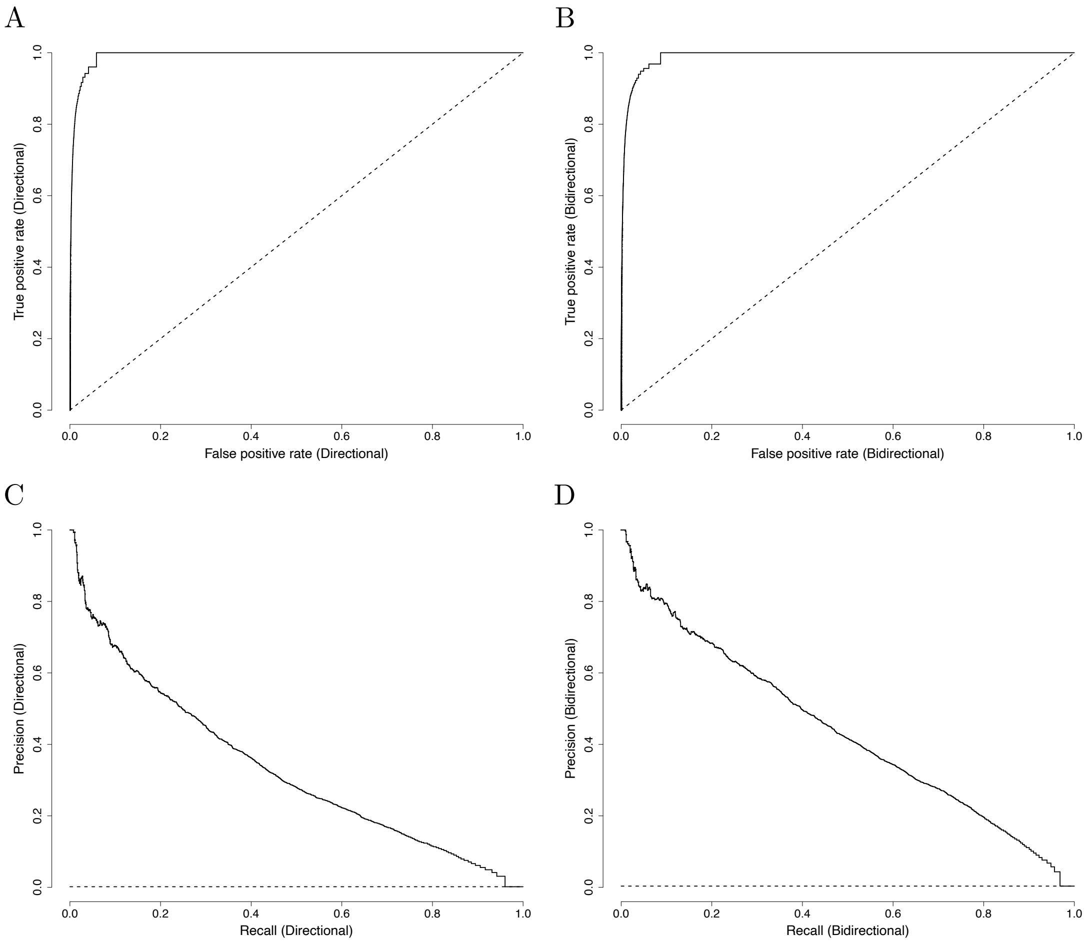

Figure S3: Receiver Operating Characteristic (ROC) curves for directional (A) and non-directional (B) transmission links averaged across the 50 benchmarking simulations. Precision-Recall (PR) curves for directional (C) and non-directional (D) transmission links averaged across the 50 benchmarking simulations.

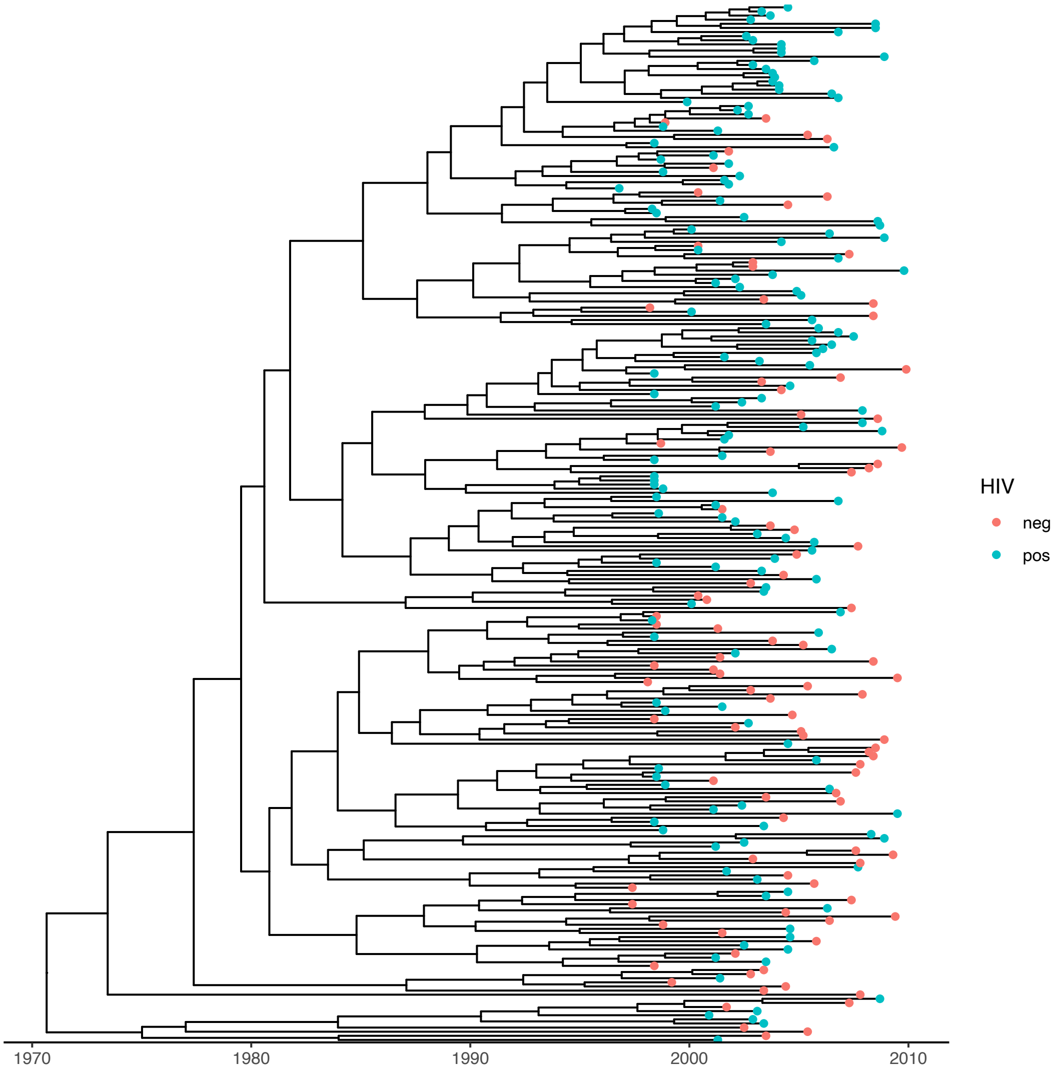

Figure S4: Dated tree used in the tuberculosis application, with leaves coloured by HIV status.

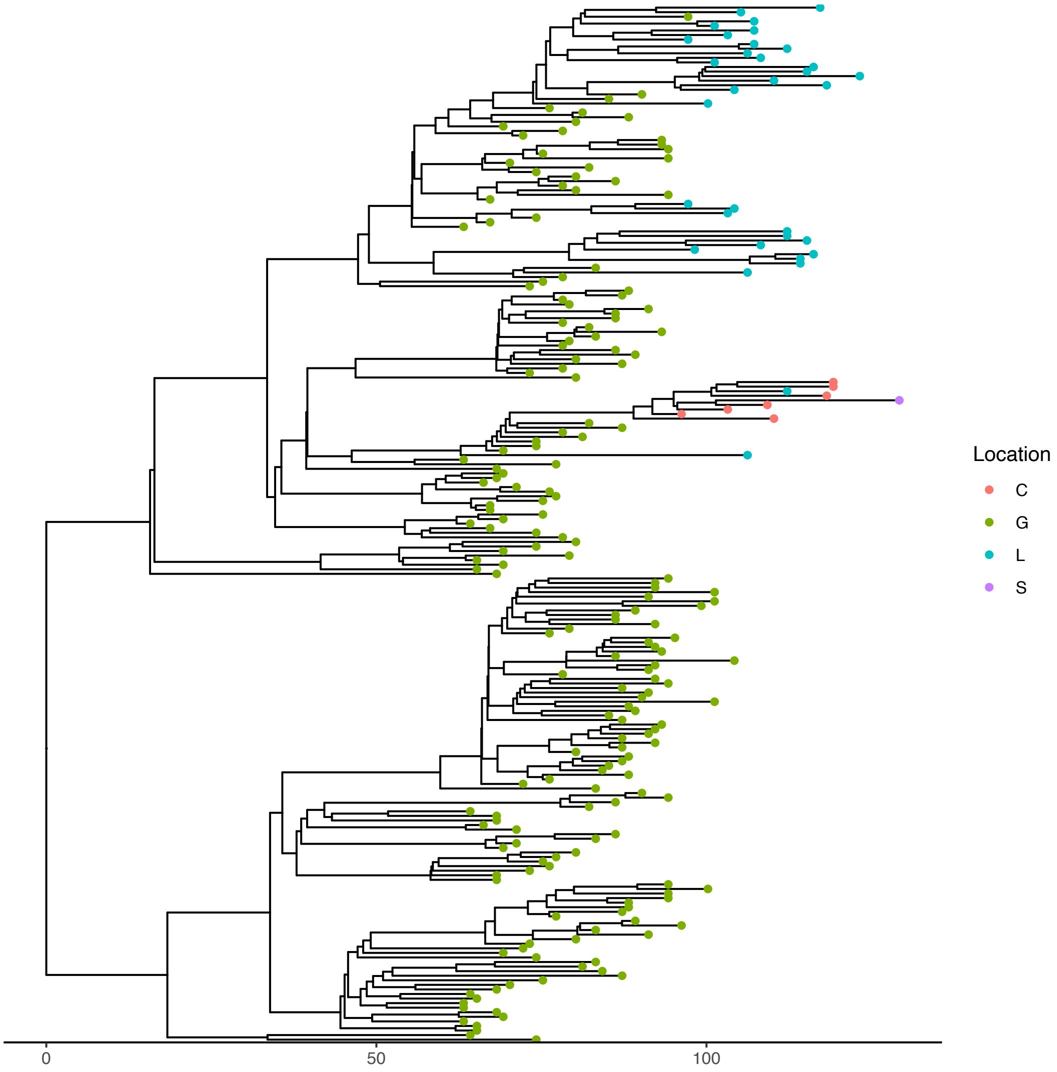

Figure S5: Dated tree used in the H7N7 application, with leaves coloured by location.

A

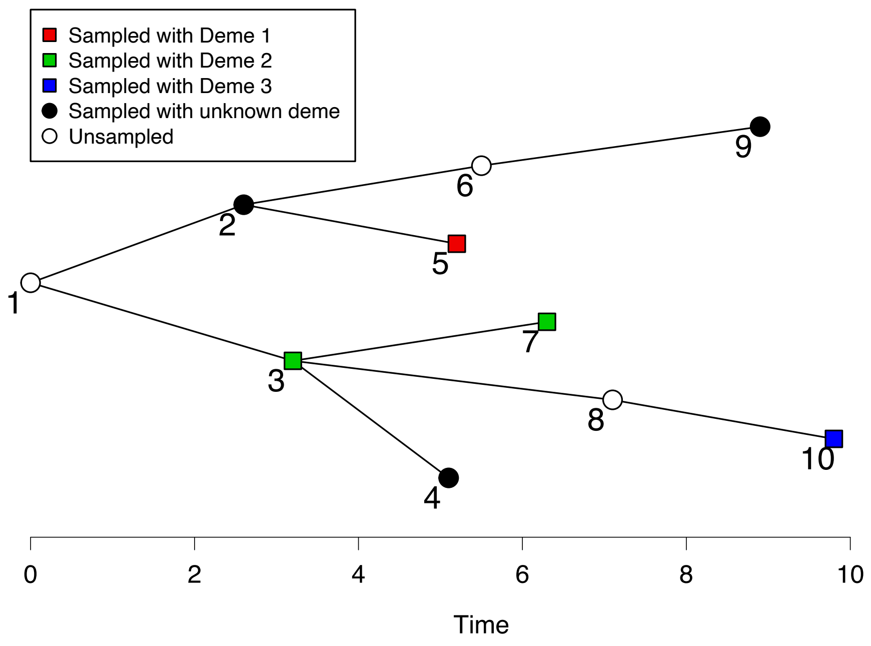

B

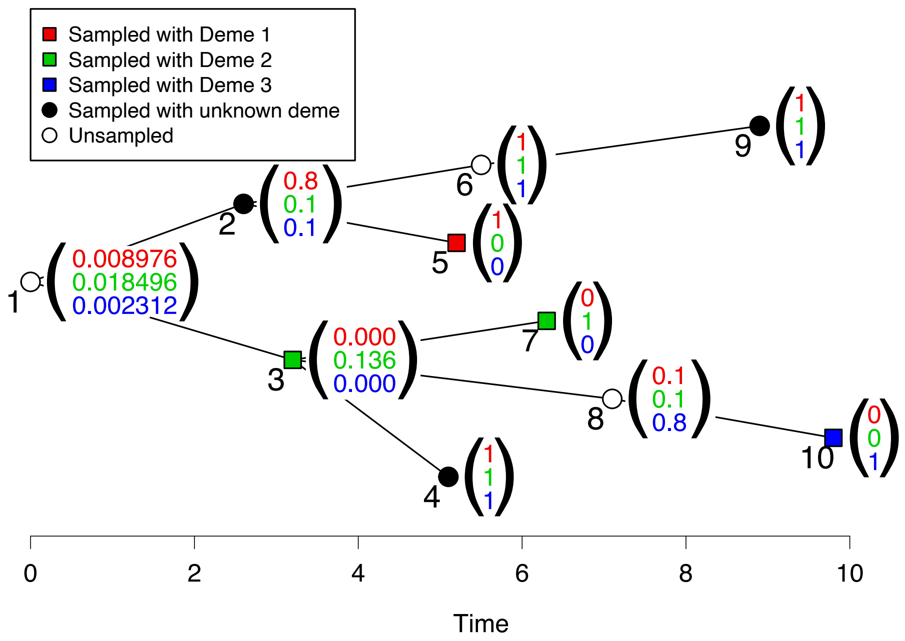

Figure S6: (A) Example transmission tree with 10 hosts. Three hosts are unsampled, three hosts are sampled without any deme data, and four hosts are sampled with deme data. (B) Conditional likelihoods in the dynamic programming algorithm with three demes, shown as vectors by each host representing the conditional likelihood at the three demes. The final likelihood for the demes is the mean of the three values for Host 1.

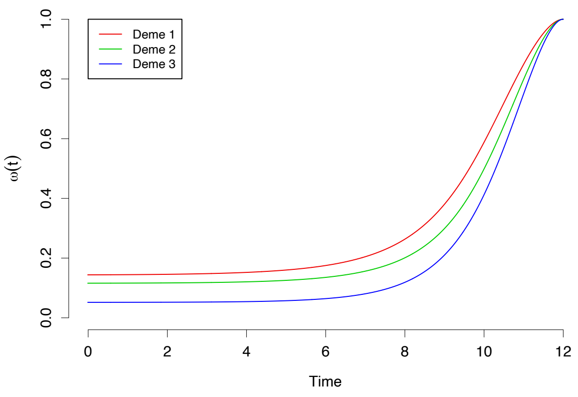

Figure S7: Exclusion probabilities through time for the illustrative example.
